# Family Migration and Social Integration of Migrants: Evidence from Wuhan Metropolitan Area, China

**DOI:** 10.3390/ijerph182412983

**Published:** 2021-12-09

**Authors:** Yanan Li, Chan Xiong, Zhe Zhu, Qiaowen Lin

**Affiliations:** 1 Business School, University of Shanghai for Science and Technology, Shanghai 200093, China; lyn@usst.edu.cn; 2 College of Management, Wuhan Institute of Technology, Wuhan 430205, China; zhuzhe9427@163.com; 3 School of Economic and Management, China University of Geosciences, Wuhan 430074, China; linqiaowen@cug.edu.cn

**Keywords:** family migration, social integration, migrants, Wuhan metropolitan area

## Abstract

The social integration in host cities of China’s migrant population and its determinants has received much attention from researchers. However, few have directly addressed the family migration in differentiating migrants’ social integration. This study employs descriptive statistics and multivariate regression to explore the social integration across different family migration types, based on the data of China Migrants Dynamic Survey 2013 and 2017 in Wuhan metropolitan area. The findings show that the family migration in Wuhan metropolitan area is prevalent, and the central city Wuhan has advantages both in the proportion of whole-family migration and the scores of migrants’ social integration. In addition, the migrants’ family migration is significantly positively associated with their social integration, but the effect is variance in the regression models of social integration’s four dimensions. The findings reveal the Wuhan City’s leading position in promoting migrants’ social integration within the Wuhan metropolitan area. Furthermore, migration with more family members has higher levels of integration in economy and public service. This implies that the convenience provided by inflow cities’ government for family migration is crucial. To provide high-quality public services is of great significance to attract and retain migrants and then promote their overall social integration.

## 1. Introduction

Over the past four decades or so, China has experienced a rapid urbanization process with numerous migration flows searching for job opportunities. According to the key data of the seventh nationwide population census released by the National Bureau of Statistics (NBS), the number of migrants has reached almost 380 million in 2020 [1]. The continuous influx of migrants greatly promotes the urban construction and economic development of host cities, as well as their own economic situation. However, the social integration of migrants remains problematic, which not only affects the migrants’ psychological health, but also erodes the host cities’ social cohesion and social stability. Therefore, improving the sizeable and ever-increasing migrants’ social integration has become a crucial societal aspiration [2]. In 2014, China released the New Urbanization Plan to carry forward the people-oriented new-type urbanization, which means the government has noticed the social segregation of migrants and tried to achieve their smooth settling in urban society formally [3]. Therefore, it is particularly important to understand the migrants’ social integration and the factors that shape their integration status. 

Research on social integration has explored its concept connotation and numerous influencing factors, such as individual and institutional characteristics [4,5]. The family-based perspective is scarce. Only in a limited amount of research do the authors mention that the family migration is especially relevant to the migrants’ settling down and assimilation into the urban society [6]. Family migration, as a senior form of migration, has been under researched in factor studies of migrants’ social integration. In the last decade, the spouse and children joint migration pattern is quickly replacing the sole and couple migration patterns that were prevalent among earlier, older migrants and cohorts [7]. On one hand, this trend deeply reflects the new developmental stage of China’s migration. One the other hand, the family migration implies entirely different conditions in all areas of life, which involve socio-economic capital, public service demand, interpersonal environment, and communicative needs. Further, the migrants’ social integration is very likely to be affected. Evidence from Western countries has indicated that international migrants’ integration outcomes have been influenced by delays in family reunification, based on micro-level data from Europe, USA, and Canada [8]. Regrettably, previous studies on migrants’ social integration in China have preponderantly focused on individual-based factors, and few studies explore the underlying mechanism of family migration’s influence on migrants’ social integration, both in theoretical and empirical analysis.

This study aims to shed light on the effect of family migration on migrants’ social integration, which is conductive to policy implications committed to improving migrants’ integration in host cities. Meanwhile, the paper tries to investigate the empirical evidence to verify the influence mechanism using data from a metropolitan area sample. The remainder of the article is arranged as follows. Section 2 is a review of studies on the Chinese migrants’ family migration, the migrants’ social integration and its dimensions are also provided. Section 3 presents the design for the empirical analysis, including data and method. Section 4 explains the empirical findings and robustness check. The discussion and conclusion are reported in the last sections.

## 2. Literature Review

### 2.1. Family Migration of China’s Migrants

According to the existing literature, a consensus has been reached that the family migration feature has become one of the major trends among China’s migrant population. At the beginning of the reform and opening-up, most of the migrant workers are singles or married men, who are working for a living while leaving the family members behind [9]. During 2000 to 2010, more family members are involved in the migration, and data from the 2011 Migrant Dynamics Monitoring Survey also shows that almost 70% of the migrant population residing with their family members [10]. Recent research provides evidence that the sole migration account for only 24.0% based on a national sampling survey conducted in 2017 [11].

Nonetheless, the definition and classification of family migration vary in current studies, mainly because the different understanding of “family” and diverse data sources [12,13]. A typical example is “marriage & kinship family” theory, that is, the family migration means more than one family member (spouse or consanguinity) lives together in the host city [14]. Based on manifold categories of “family migration”, scholars give their estimation of the development degree and main trend of family migration, respectively [7,10,14,15]. Moreover, the process of family migration has received scholars’ attention. Some researchers argue that the migration of a family is mostly incremental, which means there are pioneer and follower generally, and the batch interval is getting shorter [16,17]. Besides, the influencing factors of family migration has also been examined [18,19,20,21,22].

As a factor, the family migration has been stressed in research on determinants of migration decisions [23,24,25,26] and settlement intention [27,28,29]. However, only a few researchers pay attention to the relationship between family migration and social integration, such as a research on family migration in China suggests that as a senior form of migration, the family-based population shift itself is an important aspect of the social integration [6]. Especially, the effect of “family migration” on migrants’ social integration is still largely ignored. Apart from the migration pattern’ significance in shaping migrants’ behavior custom, empirical studies in Western countries has confirmed the impact of delays in family reunification. In Chinese context, understanding family migration’s effect on social integration is of great significance as well, which is beneficial to China’s policies regulating migration.

### 2.2. Migrants’ Social Integration and Its Dimensions in China

China has the world’s largest scale of internal migrants. The continuous influx and rapid growth of migrant population have increased the migration-related challenges. Along with the Chinese central government’s people-centered urbanization strategy, migrator’s social integration has become an important policy and academic issue. Social integration refers to a process or a degree to which people are integrated into the systems of a social structure [30]. Additionally, the major components of social integration are equal rights and opportunities in society, shared values, and trust among social systems [31]. The integration of internal migrants is often believed to be not as difficult as international migrants due to the same racial origin and broadly similar religions [32]. However, the assumption that internal migrants are more homogenous than international migrants is likely a mistake [33], especially in counties with a large population. In China, migrants, especially rural migrants, mostly differ from the locals in social, cultural, and economic features, and they generally tend to consider themselves as outsiders [34]. In a way, China’s internal migrants face similar migration-related integration challenges as many international migrants concerning their disadvantaged position.

Research on international immigrants’ integration in Western countries has generated several theories, such as the classical assimilation theory introduced by the Chicago school and the segmented assimilation framework [3,5]. Against the backdrop of international immigration integration studies, scholars try to conduct a multidimensional deconstruction of social integration in conceptualization and measurement [35,36]. Although there are no consistent terms on social integration’s dimensions, four aspects have been primarily adopted to measure the social integration: social relations, economy, psychology, and culture [32,37]. Some other dimensions, such as living condition, physical condition, personal safety, life satisfaction, and confidence in life, have also been estimated in a literature [38]. Considering China’s unique social–economic environment and central government’s integration-promoting policy orientation, recent studies emphasize the politics and public service dimensions, which including citizenship, political participation, employment rights, children’ basic education, sense of belonging, etc. [39,40].

Scholars argue that the Chinese migrants are experiencing formidable institutional, geographical, economic, cultural, and social barriers during the process of social integration [4,41]. Despite the reforms have extended the coverage of basic welfare to migrant population in the last few years, the household registration system (*Hukou*) is still regarded as a vital institutional factor hindering the process of migrators’ integration into cities [42]. Hence, research on the influential factors of migrants’ social integration has explored numerous factors, such as individual characteristics, institutional arrangements, social networks [43,44,45]. Individual factors, for example, gender, age, marriage, income, and length of stay, generally affect migrants’ social integration. Scholars have also demonstrated the important role of human capital in the success or failure of social integration [46]. Notably, some researchers indicate that the anti-migrant attitude held by natives would undermine migration’s integration [47,48].

Compared to factors relating to migrants’ outward network with natives, much less attention has been given to the household characteristics representing structure and relations within a migrant family. The family migration, illustrating the inward structure of family members who reside together in the inflow cities, should be discussed. Although some literature has taken household features, such as the number of children and the household size, into account without distinguishing these family members’ location [49], the completeness of migrant families cannot be illustrated. Furthermore, studies on immigrants’ integration in the West has shown evidence on the family migration’ positive role in promoting assimilation [50].

Thus, this paper seeks to extend the research by examining the impact of family migration on migrants’ social integration. The family migration is characterized by the degree of household completeness (in destination cities), which can not only underscore the household size in the host society, but also reflect the family relations. Moreover, taking the Wuhan metropolitan area as a representative case, this study introduces the inflow city characteristic in the analytical framework, scrutinizing the difference between the key city and non-central cities within the metropolitan region particularly. The study explores the differentiation of social integration among different cities and how the family migration affects migrators’ social integration, which could provide insights for policymaking and facilitate the formation of more targeted policy regarding migrants’ social integration.

## 3. Data and Method

### 3.1. Study Area

**Figure 1:**
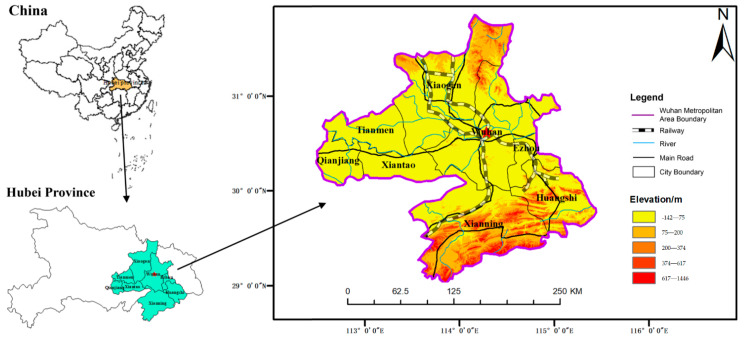
Spatial location of the Wuhan metropolitan area.

The Wuhan metropolitan area, located in the eastern part of Hubei Province and the middle reaches of the Yangtze River, is an important growth pole of the Yangtze River Economic Belt. This metropolitan area is one of the most important urban agglomerations in central China. The region includes Wuhan, the provincial capital city, and eight surrounding cities (Figure 1). Five of them are prefecture-level cities (Huangshi, Xianning, Huanggang, Xiaogan and Ezhou), and the other three are county-level cities (Xiantao, Tianmen and Qianjiang). By the end of 2020, the gross local product of the Wuhan metropolitan area reached 2.63 trillion-yuan, accounting for more than 60 percent of the total GDP of Hubei Province. In the ranking of the scale of migrants, Wuhan is rising significantly, which shows that the absorption of the migrant population is increasing sharply. 

China’s central government is proceeding the coordinated development of large, medium, and small cities, taking metropolitan area as the main body. Along with this policy guidance, the importance of migration-related issues from a “metropolitan-area” perspective has been highlighted. This situation requires a more comprehensive understanding of the social integration throughout the metropolitan region.

### 3.2. Data Source

The family migration type and its influence on social integration of the Wuhan metropolitan area’s migrants will be examined. Three sets of data were used in the study. The major data was derived from the 2017 Migrant Dynamics Monitoring Survey (MDMS henceforth), collected by the National Health Commission in China. The main reason for choosing MDMS in 2017 is that the survey has focused on the social integration issue especially. Additionally, this issue has just been involved partly in MDMS in 2014, 2015, 2016, and 2018. MDMS 2017 uses the three-stage stratified PPS (probability proportional to size) sampling, and respondents include the people aged 15 years and above who live in the city for one month or more without local household registration. The survey mainly focuses on respondents’ personal and family information, migration status, employment characteristics, social activities, health status, living conditions, children’s education, etc. The total sample size is 2600, including 2000 observations for Wuhan and 600 observations for other cities. The data of Wuhan metropolitan area excludes Qianjiang, as the survey does not include Qianjiang in 2017. This set of data was used for the statistical analysis of family migration and social integration, and more importantly for the regression analysis of family migration’ influence on social integration.

The second set of data, a special sub-survey of MDMS in 2013, was collected to assess the changes in social integration of Wuhan’s migrant population. This survey is mainly about social integration and psychological health, and it covers eight representative large Chinese cities or districts including Wuhan. A sample of 1999 Wuhan’ migrants was obtained. In addition, the urban residents’ disposable income of sample cities was obtained from the statistical yearbooks (2017) for the measurement of economic integration as the third set of data [51].

### 3.3. Variable Selection and Measurement

#### 3.3.1. Social Integration (Dependent Variable)

- Dimensions

Given the elaboration of social integration in previous studies [32,37,38,39,40], four dimensions were adopted to assess the social integration, including economic integration, public service integration, social participation, and psychological adaption. As seen in Table 1, each dimension contains several indicators with the corresponding weight. These secondary indicators were selected mainly based on the survey questions. Additionally, there were trade-offs because of the need for comparison in different years, as the questionnaire design of MDMS in each year was adjusted according to the survey objective and changes in the migrants’ condition.

The values of each social integration dimension were calculated. Given the hierarchy of social integration, the importance of each dimension should be taken into full account in its proportion of total social integration. Therefore, Delphi’s method was used to determine the percentage of each dimension. The assessment of each dimension’s importance was based on structured interviews conducted in two rounds by ten experts. Finally, to facilitate the subsequent calculation and an easier understanding, the weights were given based on approximate average scoring results: economic integration (20%), public service integration (30%), social participation (20%), and psychological adaption (30%). Then, the weights of secondary indicators were assigned using “equally weighted method”, which means the weight of an indicator is 1/n (n is the number of indicators belong to this dimension).

- Assessment method

To assess social integration level, each indicator had been scored, and Min–Max Normalization method was used for non-dimension of indicators.

(1) $$\mathrm{For}\;\mathrm{positive}\;\mathrm{indicator},\;y_{i}={\frac{x_{i}-\underset{l\leq j\leq n}{\min}\left\{x_{j}\right\}}{\underset{l\leq j\leq n}{\max}\left\{x_{j}\right\}-\underset{l\leq j\leq n}{\min}\left\{x_{j}\right\}}}^{}$$

(2) $$\mathrm{For}\;\mathrm{negative}\;\mathrm{indicator},\;y_{i}={\frac{\underset{l\leq j\leq n}{\max}\left\{x_{j}\right\}-x_{i}}{\underset{l\leq j\leq n}{\max}\left\{x_{j}\right\}-\underset{l\leq j\leq n}{\min}\left\{x_{j}\right\}}}^{}$$

After calculating the scores of all indicators, the comprehensive score of social integrations was summed based on the weights of each indicator and each dimension. To analyze social integration from economic integration dimension effectively, per capita disposable income of urban residents for each city from Hubei Provincial Statistics Bureau was used as a benchmark to assess income level. Simultaneously, average per capita expenditure and rent/mortgage-to-income ratio for each city were used as benchmarks in the calculation.

#### 3.3.2. Influencing Factors (Independent Variables)

- Family migration type (core explanatory variable)

**Figure 2:**
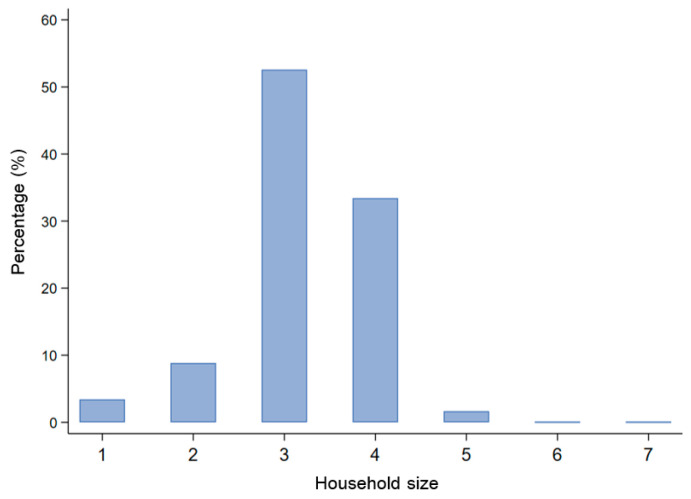
Proportion of different household sizes in Wuhan metropolitan area, 2017.

Nowadays, the phenomenon of family migration is becoming more and more prevalent, and this profile is backed by national data of CMDS in the last several years. In 2017, the average household size of national sample reached 3.14, and that of Wuhan metropolitan area was 3.21. More specifically, the three-person and four-person households’ modes accounted for more than four-fifth of all migrants in Wuhan metropolitan area (Figure 2). In the questionnaire, the “household size” refers to the number of family members who live together in inflow places from the broad sense. The family members here may be the respondents’ nephew, sister-in-law, or grandfather, who are not the immediate family members.

**Figure 3:**
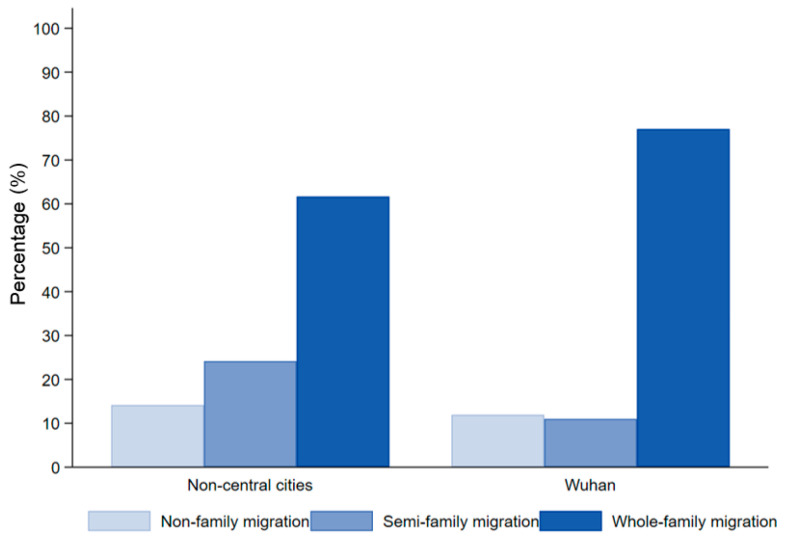
Proportion of different family migration types in Wuhan and non-central cities, 2017.

To describe the family migration accurately, we combed this concept further and identified the “spouse & unmarried children” as the core family members. That is, the “family” defined in this study includes “parents and unmarried siblings” for unmarried respondents, and includes “spouse and unmarried children” for married respondents, which means these family members of the respondent form a nuclear family. Following previous studies [10,19], the family migration was categorized by the integrated degree of nuclear family members. We identified three types for family migration analysis. The situation that all nuclear family members have completed the migration is regarded as whole-family migration; the semi-family migration refers to that two or more nuclear family members have completed the migration, but there are missing nuclear family members; only one family member migrating is non-family migration. According to CMDS2017, the proportion of whole-family migration in Wuhan has exceed 75%, which may promote the social integration of migrants. Notably, the percentage of whole-family migration in Wuhan is higher than that in non-central cities, but the percentage of semi-family migration is comparatively lower in Wuhan compared with in non-central cities (Figure 3). The chi-squared test was conducted to assess the statistical difference between Wuhan and non-central cities. The chi-square value was 73.6754 and the *p* value was close to 0, demonstrating the significance of difference. Additionally, the statistics showed that the whole-family migration accounted for the highest proportion with the size of 1911, including 370 in non-central cities and 1541 in Wuhan City. The other sample sizes were reported in Appendix (See Appendix A).

- Other explanatory variables

Besides family migration type, this research also selected a series of explanatory variables that are likely to play a role in shaping different levels of social integration. In detail, five groups of variables were chosen as explanatory variables: individual characteristics, including gender, age, age square, marital status, education level, political status, family scale and health status; institutional characteristics, i.e., household registration attribute (*hukou*); mobility characteristics, including duration of migration, range of migration, and destination city; and employment characteristics, i.e., employment status, working hours, and housing characteristics. The age was centralized by removing its mean from the data in the analysis. In addition, industry was selected as a control variable to improve the reliability of the model.

Table 2 summarized the descriptive statistical analysis of the sampled migrants. About 48% of the respondents are male, and the mean age is about 35 years old. A clear majority (91%) of the migrants are married, which is consistent with their mean age. The respondents tend to have low education levels, with about 73% having reached the middle school and high school level. Only 249 respondents are communist party members or league members, reflecting the features of their political identity. The respondents’ average family scale is 3.2, which is very close to the national average. Additionally, the result of self-rated health is quite good, with 78% consider themselves “healthy” and 20% consider themselves “basically healthy”. The migrants who have the household registration of city are in the minority (19%). With respect to family migration, a relatively high proportion of migrants realize whole-family migration (74%). In contrast, the percentages of migrants in a semi-family migration or non-family migration are almost equal, and both are much lower than that in a whole-family migration condition. Furthermore, 77% of the respondents come from Wuhan city and 23% from other non-central cities of the metropolitan area.

The proportion of employees among the migrant population is quite inconsistent with people’ common perception, which is only 31%. This is in connection with the division of groups in our research. Further, the answer to the survey question “What is your employment status?” provided 5 options: 1, employee with fixed employer; 2, employee with unfixed employer; 3, employer; 4, self-employed worker; and 5, other. As the first two groups are specific employees, we classified them as the “employee” group. The third and fourth groups are classified as one category (“other” group) out of an understanding that the self-employed worker is another form of employers, in a sense. Additionally, the last group is also put into the “other” group. Need of special note is that the “self-employed workers” are in the majority: the sample size of “self-employed worker” is 1093 and that of “employer” is only 187. In sum, we used “employee” to include the first and second group, and “others” to include the last three groups, i.e., employer, self-employed worker and other. Additionally, more than half of the respondents (54%) are living in the rented house, with 33% living in the self-purchased house.

### 3.4. Model

Regression analysis can be used to study the cause–effect relations between two or more variables. In this research, we constructed five multiple linear regression models to analyze the relationship between variables as below.

(3) $$Y_{i}=\alpha +\beta_{1i}X_{1i}+\beta_{2i}X_{2i}+\beta_{3i}X_{3i}+\beta_{4i}X_{4i}+\cdots +\varepsilon_{i}$$

$Y_{i}$ (*i* = 1, 2, …, 5) is the explained variable for the *i*-th model. $Y_{1}$ is the total score of social integration, and $Y_{2}-Y_{5}$ are scores of social integrations from dimensions of economic integration, public service integration, social participation, and psychological adaption, respectively. $\beta_{ji}$ is the estimated regression coefficient of the *j*-th variable, $X_{ji}$ is the explanatory variable, and ε is the stochastic disturbance.

## 4. Empirical Analysis

### 4.1. Comparison for Social Integration

#### 4.1.1. Wuhan and Non-Central Cities

**Figure 4:**
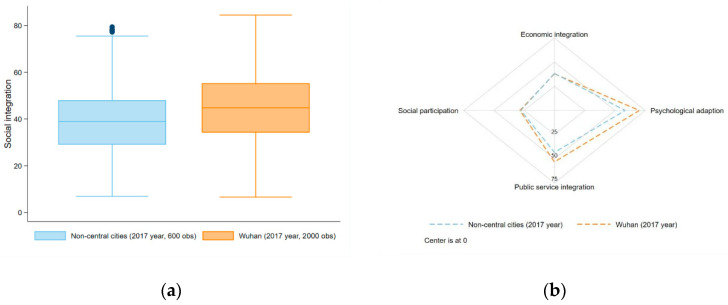
(**a**) Boxplot of Wuhan and non-central cities in terms of migrants’ social integration in 2017; and (**b**) scores of Wuhan and non-central cities in terms of social integration’s four dimensions in 2017.

In 2017, the overall level of social integration in Wuhan is higher than that in other non-central cities (Figure 4a). In terms of the four dimensions of social integration, the average levels of social integration for economic integration and social participation in Wuhan are not significantly different from that in other cities, but the average levels of social integration in Wuhan for public service integration and psychological adaption are obviously higher than that in other cities (Figure 4b). The *t* test was conducted to understand differentials between the migrants of Wuhan City and non-central cities in social integration (See Appendix B). Four dimensions and the overall score of social integration were examined, and results showed that all the mean values in Wuhan City are higher than those in non-central cities except economic integration. That indicated that the difference of integration in economic dimension between Wuhan City and non-central cities was not significant. Nevertheless, the differences in overall social integration and the other three dimensions were still statistically significant. The sample sizes were 600 in non-central cities and 2000 in Wuhan City.

#### 4.1.2. Wuhan (2013 Year and 2017 Year)

**Figure 5:**
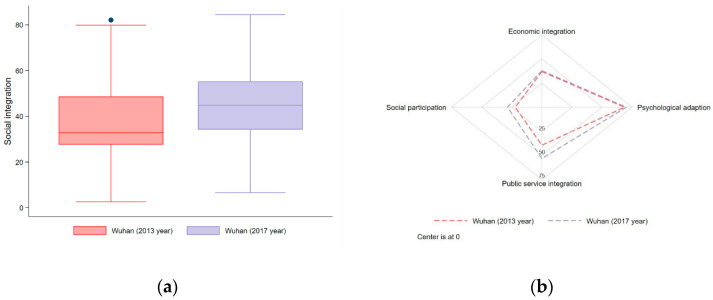
(**a**) Boxplot of Wuhan in terms of migrants’ social integration in 2013 and 2017; and (**b**) scores of Wuhan in terms of social integration’s four dimensions in 2013 and 2017.

We used the same methodology to calculate the 2013 score of social integration in Wuhan and compared it with that in 2017. As seen in Figure 5, the overall level of Wuhan migrants’ social integration in 2017 is significantly higher than that in 2013, especially for social participation and public service integration, which may be caused by the development of society and economy, and the improvement of public policies. The *t* test results in Appendix C demonstrated that the social integration significantly increased from 2013 to 2017 in Wuhan City. Additionally, the sample sizes were 1973 in 2013 and 2000 in 2017.

### 4.2. Correlation Analysis

We used Pearson correlation test (See Appendix D) to study the relationship between variables. Based on the results, variables education level, health status, household registration attribute, family migration, duration of migration, range of migration, destination city, employment status, and self-purchase house are significantly positively correlated with social integration. Variables working hours, renting, and other housing type are significantly negatively correlated with social integration.

### 4.3. Regression Analysis

This study had constructed five regression models. Additionally, the multicollinearity test and the heteroscedasticity test on the preliminary models also been conducted.

#### 4.3.1. Multicollinearity Test

Multicollinearity occurs when explanatory variables are correlated in a regression model, which will reduce the precision of the estimated coefficients. For this study, we assessed multicollinearity using VIF (Variance Inflation Factors).

VIF is a traditional indicator that can be used to detect the degree of multicollinearity in regression analysis. The VIF for the *i*-th explanatory variable is:

(4) $$VIF_{i}=\frac{1}{1-R_{i}^{2}}$$

Here, $R_{i}^{2}\;$is the $R^{2}$ value calculated by regressing the *i*-th explanatory variable on the remaining variables.

Based on the calculation methodology of VIF, variables’ VIF values for our five models are consistent. As seen in Table 3, all VIF values are less than 5, which means there is no significant multicollinearity problem among independent variables in our models.

#### 4.3.2. Heteroscedasticity Test

In linear regression analysis, if the residuals of the model are not homoscedastic, the estimated coefficients using OLS (Ordinary Least Squares) will be not reliable. In this research, we used Breusch–Pagan and White heteroscedasticity tests to check whether residuals were heteroscedastic.

For White heteroscedasticity test, we needed to fit a new regression model using the squared residuals of our current regression model as the explained variable, original explanatory variables, the square value of original explanatory variables, and the cross-products of original explanatory variables as the new explanatory variables.

(5) $$e_{i}^{2}=\alpha_{0i}+\alpha_{1i}X_{1i}+\alpha_{2i}X_{2i}+\alpha_{3i}X_{1i}^{2}+\alpha_{4i}X_{2i}^{2}+\alpha_{5i}X_{1i}X_{2i}\ldots +\varepsilon_{i}$$

Then, we calculated$\;R^{2}$, and $nR^{2}\sim \chi^{2}$, which is a Chi-Squared test. If the *p*-value is less than 0.05, we should reject the null hypothesis and conclude that heteroscedasticity is present in the regression [52]. Breusch-Pagan heteroscedasticity test is similar with White heteroscedasticity test, but it does not include the square value and the cross-products of original explanatory variables in the squared residuals’ regression model [53].

According to the results of Breusch–Pagan and White heteroscedasticity tests (Table 4), the *p*-values are both less than 0.05, we rejected the null hypothesis at 0.05 level, which means the model has heteroscedasticity problem. Therefore, we used robust option in Stata for estimating the standard errors and adopted the Huber–White sandwich estimators to deal with issues in the model caused by heteroscedasticity.

#### 4.3.3. Results of Regression

The empirical results of the multivariate regression were presented in Table 5. All the models were updated with adjustments. This study measured the effect of family migration on migrants’ social integration in model 1 to model 5, where dependent variables were scores of the overall social integration, economic integration, public service integration, social participation, psychological adaption, respectively.

Overall, migrants’ family migration is significantly positively associated with their social integration. However, the estimates of four dimensions of social integration show widely different results. Interestingly, the family migration is not statistically related to social participation and psychological adaption, which set us thinking. Although this result is inconsistent with our expectations, it has reflected the complexity of the integration issue. By contrast, the family migration contributes to a significant impact on migrants’ economic integration and public services integration. Yet, the former effect is negative, and the latter effect is positive. Migrants with more family members, especially those preschool children, students, elderly parents, and disabled people, would greatly increase the living cost. This will undermine the capability of migrants’ economic integration. As for the family migration’s positive association with public services integration, one possible explanation is that the increase of immediate family members in destination cities fueled the demand for public services. Or rather, the migrants who are entitled to receive better public services are more inclined to migrant with immediate family members. In summary, the public service is the core factor in the consideration of whether to adopt a non-split household strategy. However, the family migration does not necessarily imply a permanent settlement intention (indexes included in psychological adaption), it’s more like a phased decision after cost–benefit calculation of the entire household’s gains.

In terms of individual characteristics, as seen model 1 in Table 5, women have a higher level of social integration than men. The age has a non-linear effect on social integration, and the inflection point is around 45. Before the age of 45, with the increase of age, the level of social integration of people increases, and after reaching the highest point near the age of 45, the level of social integration of people gradually decreases. This may be due to the middle-aged people have higher income levels, more leisure time, and social needs. People who have better education background have a higher level of society integration, since they have a stronger ability to integrate into society. In addition, healthier people have a higher level of social integration. At the same time, people with urban household registration have a higher level of social integration. The most important reason may be that people with urban household registration and people in the destination city have similar living habits and cultures.

For household characteristics, as outlined above, the degree of family migration influences the social integration significantly. People who realize complete family migration have a higher level of social integration. For mobility characteristics, the longer people move to the destination city, the better they integrate into it. It is due to social integration often takes time. Additionally, people who migrate across provinces can better integrate into the society in general, despite the significant negative estimator of parameter in psychological adaption model. This phenomenon may attribute to their destination city. The destination locations of cross-province migration are, for the most part, Wuhan. According to statistics, 75% of the cross-province migrants reside in Wuhan, where having better job opportunity and social welfare, which can promote the access to public resources and create their advantages in public services integration. That is in line with the regression result of “destination city” factor: the overall level of social integration in Wuhan is higher than that of other non-central cities. This may be due to the better public services and the unique city charm of Wuhan, the central city.

Moreover, employees with shorter working hours can better integrate with society since they have more leisure time and vigor. For housing characteristics, people who live in their own houses have a higher level of social integration than others, since they have a stronger sense of belonging to this city. In summary, the regression results of model 1 are basically consistent with our expectations.

For model 2, since most married people have children, their per capita income are often affected, and the level of economic integration is also affected by the family scale as well. People with higher degree often have higher income and consumption levels. In the meantime, people with their own business always have higher income. The results of model 3 are very similar with that of model 1. In model 3, the level of social integration in Wuhan is significantly higher than that of other non-central cities. It may be due to Wuhan have better public services and institutions. For model 4, men are more willing to participate in social activities. People with better education background often have more social needs. Additionally, long working hours directly affect people’s participation in social activities negatively. According to the results of model 5, people with urban household registration have a higher level of psychological adaption since they have similar cultures and backgrounds with those people in the destination city. Simultaneously, people who migrate from a closer county or city have similar cultures and even dialects with the people in the destination city. Wuhan has a higher level of social integration in the perspective of psychological adaption. This may be because Wuhan, as a metropolis, can better embrace non-natives and other cultures.

### 4.4. Robustness Check

The propensity score-matching method (PSM) was commonly used to reduce the bias in the estimation of treatment effects with observational datasets [54]. The propensity score was defined as the conditional probability of receiving a treatment given pretreatment characteristics [55]. In this study, the PSM acted as a solution to the endogenous problem and was employed as a robustness check to compensate for the deficiency of the OLS method.

In the PSM processing, the gender and age variables were used as grouping variables, and matched samples were used for re-testing with sample size 1755 and 1089, respectively. As the age was not a dichotomous variable, it was grouped in “strong working-ability sample” and “weak working-ability sample”, which is one group aged from 30 to 50 and the other group aged under 30 and above 50. Specifically, we used a logit model to estimate the regression coefficients of migrants’ explanatory variables related to social integration. The parameters of PSM were non-replacement sampling, 1:1 pairing, and 5% significance level. The results of PSM in Table 6 and Table 7 reported that the findings were slightly different from those of OLS. The directions and significance levels of core variables’ effect were generally consistent with the original analysis. That is, after solving the endogenous problem, the same conclusion can be obtained using the matched samples, so the results were robust.

## 5. Discussion

Using multivariate regression model and data from 2013 and 2017 MDMS, this paper investigated the migrants’ social integration and its determinants in Wuhan metropolitan area. We especially focused on the role of migrants’ family migration in shaping their social integration and its four dimensions. This study contributed to the literature in three ways. First, it introduced the family migration into the analysis framework of social integration research. Second, it distinguished between city attributes within a metropolitan area. Third, it adopted social integration’s four dimensions to examine the influencing factors (i.e., economic integration, public services integration, social participation, and psychological adaption), as well as the overall social integration. Our findings remain robust after controlling for potential endogeneity bias.

The findings clearly revealed the status of family migration and social integration in the Wuhan metropolitan area, and its difference between Wuhan and non-central cities. The average household size of the Wuhan metropolitan area has reached 3.21, and the percentage of whole-family migration in Wuhan is higher than that of non-central cities. Additionally, the score of overall social integration in Wuhan is higher than that of other cities in the metropolitan area as well. The study also depicted the changes of overall social integration and its four dimensions between 2013 and 2017 in Wuhan city. Wuhan city, as the central city of the metropolitan area and the capital city of Hubei province, has shown enormous advantages and potential in supporting migrant population’s public services sharing and long-term residence.

Our research confirmed a positive impact of family migration on social integration. Several other factors were also highlighted. Such as the female and better educated migrators are more integrated into the host society, and the older the higher level of social integration before the age of 45. The results of these factors are mostly consistent with existing studies.

## 6. Conclusions

This study has investigated how family migration affects social integration of China’s migrants. Except for effects from household features, the existing literature hardly offers conclusive results on this question. Due to data limitations and methodological challenges, empirical results are difficult to obtain. The comparative analysis on social integration was only conducted in Wuhan City. In order to separate causal effects of family migration from spurious correlations, a series of other factors were taken into account. The PSM approach has been employed to solve endogenous problems and for robustness check as well.

The results indicated that the higher level of family migration, the better social integration of migrants in host destination generally. The variance of model regression results in social integration’s four dimensions gives an index to the underlying mechanism of the family migration’s role in determining their social integration. That is, a migrant’s choice between split-household arrangement or whole-family migration is, to some degree, a tradeoff decision after weighing the income, expenses, social benefits and sentimental value from a family perspective. Additionally, the family migration’s effect on social participation and psychological adaption in the inflow cities is not significant. That inspired us to demonstrate the importance of improving the migrants’ public service in the host society, rather than emphasize the social activities and expand their social circles simply. That is, to facilitate the migrants’ family migration is of great significance to attract and retain migrants and then promote their overall social integration.

In the region of Wuhan metropolitan area, the central city Wuhan has significant advantages both in family migration degree and social integration. With regard to the Chinese government’s coordinated development policy, i.e., taking metropolitan area as main body, the central city’s dominant and leading position should be further encouraged and supported.

## Figures and Tables

**Table 1:** Dimensions and indicators of migrants’ social integration.

Dimension	Indicator
Economic integration (20%)	Per capita monthly income (33.33%)
	Per capita monthly expenditures (33.33%)
	Rent/mortgage-to-income ratio (33.33%)
Public service integration (30%)	Medical insurance (25%)
	Personal social insurance card (25%)
	Temporary residential permit/residential permit (25%)
	Healthcare record (25%)
Social participation (20%)	Participate in political activities (33.33%)
	Participate in activities of social organizations (33.33%)
	Social circle (33.33%)
Psychological adaption (30%)	Willingness to integrate into local society (25%)
	Willingness to settle down (25%)
	Willingness to stay in the future (25%)
	Estimated time to stay (25%)

**Table 2:** Definition and descriptive statistical analysis for variables.

Explanatory Variables	Definition	Sample Size	Sample Size	Mean	Minimum	Maximum
Individual characteristics						
Gender	Male = 1; Female = 0	2600	1264	0.5	0.0	1.0
Age		2600	2600	35.2	15.0	75.0
Age square	Age * Age/100	2600	2600	1336.1	225.0	5625.0
Marital status	Married = 1; Unmarried = 0	2600	2374	0.9	0.0	1.0
Education level	No schooling = 1	2600	26	3.6	1.0	7.0
	Primary school = 2		211			
	Middle school = 3		1176			
	High school = 4		713			
	College degree = 5		315			
	Bachelor degree = 6		142			
	Postgraduate degree = 7		17			
Political status	Communist party member/League member = 1; Others = 0	2600	249	0.1	0.0	1.0
Family scale		2600	2600	3.2	1.0	7.0
Health status	Cannot take care of themselves = 1	2600	3	3.8	1.0	4.0
	Unhealthy = 2		56			
	Basically healthy = 3		516			
	Healthy = 4		2025			
Institutional characteristic						
Household registration attribute (*hukou*)	Agricultural *hukou* = 0; Others = 1	2600	495	0.2	0.0	1.0
Household characteristic						
Family migration	Non-family migration = 1	2600	324	2.6	1.0	3.0
	Semi-family migration = 2		365			
	Whole-family migration = 3		1911			
Mobility characteristics						
Duration of migration (month)		2600	2600	77.8	2.0	444.0
Range of migration	Cross-county migration = 1	2600	532	2.1	1.0	3.0
	Cross-city migration = 2		1372			
	Cross-province migration = 3		696			
Destination city	Wuhan = 1; non-central cities = 0	2600	2000	0.8	0.0	1.0
Employment characteristics						
Employment status	Employee = 1; Others = 0	2600	809	0.3	0.0	1.0
Working hours (per week)		2600	2600	49.4	0.0	99.0
Housing characteristics						
Renting	Renting = 1; Others = 0	2600	1408	0.5	0.0	1.0
Self-purchased house	Self-purchase = 1; Others = 0	2600	864	0.3	0.0	1.0
Other housing type	Non-renting & Non-self-purchase = 1; Others = 0	2600	328	0.1	0.0	1.0

Note: If values only contain 0 and 1, the column “Sample size for each value” only includes sample size when value is equal to 1.

**Table 3:** Test of multicollinearity.

	VIF	1/VIF
Gender	1.245	0.803
Age	2.367	0.422
Age square	1.883	0.531
Marital status	1.439	0.695
Education level	1.607	0.622
Political status	1.206	0.829
Family scale	1.302	0.768
Health status	1.132	0.883
Household registration attribute	1.176	0.851
Family migration	1.202	0.832
Duration of migration	1.266	0.79
Range of migration	1.114	0.898
Destination country	1.099	0.91
Employment status	1.68	0.595
Working hours	3.649	0.274
Self-purchased house	1.259	0.795
Other housing type	1.244	0.804
Mean VIF	1.749	

**Table 4:** Test of heteroscedasticity.

Model	Test	Breusch-Pagan Test	White’s Test
Model 1	chibar2	65.42	535.53
	Prob.	0.0019	0.0002
Model 2 (Economic integration)	chibar2	91.41	776.27
	Prob.	0.0000	0.0000
Model 3 (Public service integration)	chibar2	62.24	497.46
	Prob.	0.0043	0.0095
Model 4 (Social participation)	chibar2	62.15	542.97
	Prob.	0.0044	0.0001
Model 5 (Psychological adaption)	chibar2	84.87	505.18
	Prob.	0.0000	0.0049

**Table 5:** Results of multivariate regression.

Variables	Model 1	Model 2	Model 3	Model 4	Model 5
	Overall Social Integration	Economic Integration	Public Service Integration	Social Participation	Psychological Adaption
Individual characteristics					
Gender	−1.0289 *	0.0382	−2.2044 **	1.4305 *	−1.2129
	[−1.690]	[0.214]	[−2.318]	[1.784]	[−1.307]
Age	0.1619 ***	0.0064	0.2929 ***	−0.0752	0.0049
	[3.842]	[0.426]	[4.431]	[−1.288]	[0.073]
Age square	−0.0083 ***	−0.0014	−0.0139 ***	0.0017	−0.0051
	[−2.890]	[−1.313]	[−3.180]	[0.432]	[−1.195]
Marital status	−0.8258	−1.1742 **	−0.8943	−0.2719	4.8780 ***
	[−0.731]	[−2.393]	[−0.515]	[−0.163]	[2.650]
Education level	2.2902 ***	0.5300 ***	2.5194 ***	3.3632 ***	2.5187 ***
	[7.030]	[4.804]	[4.834]	[7.738]	[5.154]
Political status	−0.9308	0.3979	−2.3275	1.9306	−1.1244
	[−0.921]	[1.130]	[−1.459]	[1.379]	[−0.739]
Family scale	0.2938	−1.1140 ***	0.6549	0.6180	0.3820
	[0.733]	[−7.918]	[1.037]	[1.148]	[0.609]
Health status	1.1951 **	0.4440**	2.0940 **	−0.7508	0.4637
	[1.968]	[2.320]	[2.194]	[−0.973]	[0.501]
Institutional characteristic					
Household registration attribute	2.0236 ***	−0.0916	3.4231 ***	−0.0596	3.5642 ***
	[2.620]	[−0.388]	[2.807]	[−0.060]	[3.233]
Household characteristic					
Family migration	1.3862 ***	−0.2696 *	2.2612 ***	0.4171	0.9698
	[3.355]	[−1.802]	[3.499]	[0.731]	[1.546]
Mobility characteristics					
Duration of migration	0.0262 ***	0.0002	0.0376 ***	0.0177 ***	0.0328 ***
	[5.770]	[0.174]	[5.292]	[3.041]	[4.895]
Range of migration	2.0747 ***	0.4263 ***	3.5015 ***	−0.5573	−2.9874 ***
	[4.967]	[3.150]	[5.273]	[−1.003]	[−4.705]
Destination city	5.0684 ***	−0.6533 ***	8.4185 ***	0.7400	11.6876 ***
	[7.264]	[−2.880]	[7.614]	[0.822]	[11.705]
Employment characteristics					
Employment status	2.0724 ***	−1.0480 ***	4.1689 ***	−1.0967	0.8905
	[2.706]	[−4.246]	[3.473]	[−1.084]	[0.810]
Working hours	−0.0691 ***	−0.0054	−0.0876 ***	−0.0772 ***	−0.0670 **
	[−3.796]	[−0.911]	[−3.130]	[−3.088]	[−2.473]
Housing characteristics					
Self-purchased house	4.1847 ***	1.5605 ***	4.9622 ***	4.4764 ***	9.3468 ***
	[6.257]	[8.092]	[4.697]	[5.260]	[9.947]
Other housing type	−2.0531 **	0.8933 ***	−3.3904 **	−0.9877	0.6309
	[−2.330]	[3.459]	[−2.439]	[−0.868]	[0.445]
Industry	Controlled				
Constant	14.9455 ***	38.6176 ***	8.4259	10.8319 **	41.1099 ***
	[4.604]	[33.907]	[1.615]	[2.547]	[8.033]
Observations	2600	2600	2600	2600	2600
R-squared	0.164	0.129	0.140	0.103	0.183

Robust *t*-statistics in brackets; *** *p* < 0.01, ** *p* < 0.05, * *p* < 0.1.

**Table 6:** Robustness check (gender as grouping variable).

Variables	Model 1	Model 2	Model 3	Model 4	Model 5
	Overall Social Integration	Economic Integration	Public Service Integration	Social Participation	Psychological Adaption
Individual characteristics					
Gender	−1.385 *	0.021	−2.647 **	0.995	−2.125 *
	(−1.70)	(0.09)	(−2.08)	(0.91)	(−1.72)
Age	0.136 ***	0.006	0.229 ***	−0.016	−0.120
	(2.61)	(0.37)	(2.79)	(−0.23)	(−1.51)
Age square	−0.007 **	−0.002 **	−0.012 **	0.001	0.001
	(−2.16)	(−2.10)	(−2.23)	(0.31)	(0.11)
Marital status	−0.157	−0.596	0.425	−1.463	7.441 ***
	(−0.12)	(−1.48)	(0.21)	(−0.76)	(3.47)
Education level	2.696 ***	0.444 ***	3.189 ***	3.470 ***	2.644 ***
	(6.95)	(3.62)	(5.06)	(6.74)	(4.51)
Political status	−1.495	0.383	−2.914	0.887	−0.426
	(−1.30)	(1.10)	(−1.58)	(0.57)	(−0.25)
Family scale	0.190	−0.858 ***	0.412	0.573	0.590
	(0.39)	(−5.70)	(0.53)	(0.89)	(0.77)
Health status	1.193 *	0.430 *	2.137 *	−0.874	−0.236
	(1.69)	(1.89)	(1.91)	(−0.94)	(−0.21)
Institutional characteristic					
Household registration attribute	1.626 *	−0.103	2.751 *	−0.021	3.452 **
	(1.71)	(−0.38)	(1.82)	(−0.02)	(2.56)
Household characteristic					
Family migration	1.352 ***	−0.048	2.157 ***	0.337	0.262
	(2.83)	(−0.33)	(2.84)	(0.51)	(0.36)
Mobility characteristics					
Duration of migration	0.036 ***	0.000	0.053 ***	0.022 ***	0.047 ***
	(6.16)	(0.03)	(5.67)	(3.00)	(5.73)
Range of migration	1.817 ***	0.343 **	3.071 ***	−0.473	−2.849 ***
	(3.61)	(2.26)	(3.80)	(−0.69)	(−3.65)
Destination city	4.545 ***	−0.704 ***	7.437 ***	1.120	11.672 ***
	(5.41)	(−2.63)	(5.54)	(1.03)	(9.91)
Employment characteristics					
Employment status	1.924 **	−0.747 ***	3.778 **	−0.968	−0.017
	(1.97)	(−2.66)	(2.45)	(−0.75)	(−0.01)
Working hours	−0.085 ***	−0.008	−0.118 ***	−0.065 **	−0.083 **
	(−3.69)	(−1.07)	(−3.32)	(−1.97)	(−2.39)
Housing characteristics					
Self-purchased house	3.910 ***	1.491 ***	4.605 ***	4.241 ***	9.879 ***
	(4.78)	(6.35)	(3.55)	(4.16)	(8.69)
Other housing type	−2.080 **	1.231 ***	−3.431 **	−1.336	−1.071
	(−1.98)	(4.15)	(−2.04)	(−0.95)	(−0.63)
Industry	Controlled				
Constant	13.453 ***	37.476 ***	6.161	11.305 **	39.760 ***
	(3.48)	(28.46)	(0.98)	(2.21)	(6.54)
Observations	1755	1755	1755	1755	1755
R-squared	0.187	0.123	0.156	0.111	0.211

Robust *t*-statistics in brackets; *** *p* < 0.01, ** *p* < 0.05, * *p* < 0.1.

**Table 7:** Robustness check (age as grouping variable).

Variables	Model 1	Model 2	Model 3	Model 4	Model 5
	Overall Social Integration	Economic Integration	Public Service Integration	Social Participation	Psychological Adaption
Individual characteristics					
Gender	−1.289	0.194	−2.905 *	2.073	−0.848
	(−1.33)	(0.61)	(−1.93)	(1.53)	(−0.56)
Age	0.134 **	0.007	0.261 ***	−0.123	0.011
	(2.03)	(0.26)	(2.60)	(−1.30)	(0.10)
Age square	−0.003	−0.001	−0.009	0.011 *	−0.007
	(−0.83)	(−0.61)	(−1.42)	(1.88)	(−1.04)
Marital status	−0.426	−1.520 **	−0.349	0.439	4.189 **
	(−0.32)	(−2.44)	(−0.17)	(0.22)	(1.99)
Education level	2.936 ***	0.344 **	3.676 ***	3.308 ***	2.083 ***
	(6.33)	(1.96)	(4.91)	(5.03)	(2.80)
Political status	−0.274	0.823 *	−1.529	2.393	0.502
	(−0.23)	(1.94)	(−0.85)	(1.38)	(0.29)
Family scale	0.250	−1.132 ***	0.401	1.180	0.466
	(0.42)	(−4.61)	(0.43)	(1.42)	(0.51)
Health status	0.452	0.835*	0.464	0.033	1.808
	(0.44)	(1.96)	(0.29)	(0.03)	(1.11)
Institutional characteristic					
Household registration attribute	2.816 **	−0.110	5.039 ***	−0.925	4.330 **
	(2.47)	(−0.27)	(2.81)	(−0.59)	(2.56)
Household characteristic					
Family migration	0.830	−0.458 *	1.620 *	−0.250	−0.641
	(1.34)	(−1.79)	(1.66)	(−0.28)	(−0.67)
Mobility characteristics					
Duration of migration	0.020 ***	−0.001	0.030 ***	0.010	0.045 ***
	(2.90)	(−0.25)	(2.85)	(0.94)	(4.36)
Range of migration	2.251 ***	0.563 **	3.589 ***	−0.076	−3.951 ***
	(3.48)	(2.32)	(3.44)	(−0.08)	(−3.83)
Destination city	5.432 ***	−0.442	8.763 ***	1.315	13.267 ***
	(5.04)	(−1.02)	(5.15)	(0.91)	(8.35)
Employment characteristics					
Employment status	2.414 *	−1.164 **	4.525 **	−0.342	1.606
	(1.94)	(−2.38)	(2.35)	(−0.19)	(0.88)
Working hours	−0.059*	0.012	−0.087 *	−0.045	−0.105 **
	(−1.92)	(1.11)	(−1.88)	(−0.97)	(−2.32)
Housing characteristics					
Self-purchased house	3.808 ***	1.526 ***	3.764 **	6.220 ***	9.905 ***
	(3.60)	(4.20)	(2.28)	(4.44)	(6.44)
Other housing type	−1.704	0.740	−3.132	0.138	0.520
	(−1.23)	(1.56)	(−1.43)	(0.07)	(0.24)
Industry	Controlled				
Constant	14.457 ***	38.023 ***	10.538	2.648	42.301 ***
	(2.88)	(17.84)	(1.32)	(0.40)	(5.34)
Observations	1089	1089	1089	1089	1089
R-squared	0.192	0.125	0.162	0.124	0.217

Robust *t*-statistics in brackets; *** *p* < 0.01, ** *p* < 0.05, * *p* < 0.1.

## Data Availability

Restrictions apply to the availability of MDMS2017 and MDMS2013. The data was obtained from National Health Commission of PRC and are available at https://chinaldrk.org.cn/wjw/#/ (accessed on 6 December 2021) home with the permission of National Health Commission of PRC. Publicly available datasets were also analyzed in this study. The urban residents’ disposable income data of sample cities can be found here: http://tjj.hubei.gov.cn/tjsj/sjkscx/tjnj/gsztj/whs/ (accessed on 6 December 2021).

## Appendix A

**Table A1:** Chi-Squared Test.

City Attribute	Non-Family Migration	Semi-Family Migration	Whole-Family Migration	Total
Non_central	85	145	370	600
Wuhan	239	220	1541	2000
Total	324	365	1911	2600

chi2(2) =73.6754, *p*_value = 0.000.

## Appendix B

**Table A2:** *t* Test.

Variables	Group	Mean ± sd	*t*	sig
score	Non_central	39.21 ± 14.79	−8.58	0.00
	Wuhan	45.16 ± 14.92		
score_eco	Non_central	38.31 ± 4.99	2.10	0.04
	Wuhan	37.84 ± 4.38		
score_pub	Non_central	43.33 ± 23.12	−9.20	0.00
	Wuhan	53.15 ± 22.88		
score_soc	Non_central	27.75 ± 19.73	−0.83	0.41
	Wuhan	28.51 ± 19.11		
score_psy	Non_central	58.39 ± 23.32	−11.29	0.00
	Wuhan	70.12 ± 21.99		

## Appendix C

**Table A3:** *t* Test.

Variables	Group	Mean ± sd	*t*	sig
score	2013	32.98 ± 15.11	−25.56	0.00
	2017	45.16 ± 14.92		
score_eco	2013	33.06 ± 4.55	−33.78	0.00
	2017	37.84 ± 4.38		
score_pub	2013	36.46 ± 23.86	−22.58	0.00
	2017	53.15 ± 22.88		
score_soc	2013	22.07 ± 15.64	−11.66	0.00
	2017	28.51 ± 19.11		
score_psy	2013	68.66 ± 29.14	−1.78	0.08
	2017	70.12 ± 21.99		

## Appendix D

**Table:**

**Variables**	**Social** **Integration**	**Gender**	**Age**	**Age Square**	**Marital Status**	**Education Level**	**Political Status**	**Family Scale**	**Health Status**	**Household Registration Attribute**	**Family** **Migration**	**Duration of Migration**	**Range of Migration**	**Destination City**	**Employment Status**	**Working Hours**	**Renting**	**Self-Purchased House**	**Other Housing Type**
Social integration	1																		
Gender	0.006	1																	
Age	0.012	0.158 ***	1																
Age square	−0.067 ***	0.004	0.509 ***	1															
Marital status	0.022	−0.014	0.250 ***	−0.180 ***	1														
Education level	0.209 ***	0.055 ***	−0.359 ***	−0.183 ***	−0.114 ***	1													
Political status	0.017	−0.024	−0.204 ***	0.084 ***	−0.192 ***	0.289 ***	1												
Family scale	0.008	0.01	0.01	−0.267 ***	0.311 ***	−0.139 ***	−0.153 ***	1											
Health status	0.051 ***	0.02	−0.303 ***	−0.192 ***	−0.074 ***	0.171 ***	0.046 **	−0.025	1										
Household registrationattribute	0.123 ***	0.005	0.002	−0.012	−0.01	0.315 ***	0.109 ***	−0.073 ***	0.043 **	1									
Family migration	0.081 ***	0.046 **	0.047 **	0.011	0.207 ***	−0.072 ***	−0.043 **	0.226 ***	−0.022	−0.077 ***	1								
Duration of migration	0.124 ***	0.064 ***	0.372 ***	0.143 ***	0.013	−0.157 ***	−0.069 ***	0.083 ***	−0.139 ***	−0.007	0.100 ***	1							
Range of migration	0.046 **	−0.006	−0.034 *	−0.014	0.022	−0.085 ***	−0.059 ***	0.070 ***	0.029	0.003	0.009	−0.057 ***	1						
Destination city	0.166 ***	−0.039 **	−0.058 ***	−0.062 ***	0.032 *	0.041 **	−0.023	−0.051 ***	0.036 *	−0.079 ***	0.106 ***	−0.019	0.144 ***	1					
Employment status	0.119 ***	0.083 ***	−0.095 ***	−0.055 ***	−0.138 ***	0.211 ***	0.100 ***	−0.170 ***	0.053 ***	0.049 **	−0.152 ***	−0.105 ***	−0.144 ***	−0.003	1				
Working hours	−0.057 ***	0.273 ***	0.177 ***	−0.082 ***	0.046 **	−0.191 ***	−0.103 ***	0.086 ***	0.006	−0.072 ***	0.011	0.126 ***	0.071 ***	−0.063 ***	0.007	1			
Renting	−0.116 ***	0.027	0.011	0.012	−0.035 *	−0.194 ***	−0.084 ***	0.031	0.003	−0.144 ***	0.130 ***	−0.053 ***	0.121 ***	0.022	−0.104 ***	0.092 ***	1		
Self-purchased house	0.208 ***	−0.023	0.004	−0.013	0.076 ***	0.242 ***	0.070 ***	0.004	−0.006	0.165 ***	0.011	0.101 ***	−0.166 ***	−0.013	0.088 ***	−0.190 ***	−0.767 ***	1	
Other housing type	−0.121 ***	−0.008	−0.022	0.001	−0.055 ***	−0.051 ***	0.026	−0.053 ***	0.003	−0.019	−0.211 ***	−0.064 ***	0.055 ***	−0.015	0.03	0.130 ***	−0.413 ***	−0.268 ***	1

*** *p* < 0.01, ** *p* < 0.05, * *p* < 0.1.
